# Supplementary material for: Analysis of Survival-Related lncRNA Landscape Identifies A Role for LINC01537 in Energy Metabolism and Lung Cancer Progression
Source: Int J Mol Sci. 2019 Aug 1;20(15):3713. doi: 10.3390/ijms20153713 (PMC6696180; doi:10.3390/ijms20153713)
Supplement: Supplementary file 1 [file ijms-20-03713-s001.zip › ijms-550517-supplementary/Supplementary Figure Legends.docx]

**Supplementary Figure Legends**

**Supplementary Figure S1:** LncRNA expression profiles for TCGA lung cancer tissues or lung normal tissues. ***a-b.*** Heatmap of the differentially regulated lncRNAs in LUAD (a) and LSCC (b) from the TCGA datasets. ***c-d.*** Circos for visualization of the overlapped dysregulated lncRNAs in LUAD (c) and LSCC (d) in human genome. Blue irregular cords refer to downregulated lncRNAs and red irregular cords refer to upregulated ones.

**Supplementary Figure S2:** Bioinformatics analysis of LINC01537. ***a.*** CPATool indicated LINC01537 having no coding probability. ***b.*** Box plots show the LINC01537 expression between lung normal tissues LUAD (left) as well LSCC (right) tissues from TCGA database. ***c.*** Histogram depicts the LINC01537 expression in various cancer cell types from TCGA database.

**Supplementary Figure S3:** Validation of the expression level of LINC01537 after lentivirus infection (a) and PDE2A after shRNA treatment (b).
