# Supplementary material for: Analysis of Survival-Related lncRNA Landscape Identifies A Role for LINC01537 in Energy Metabolism and Lung Cancer Progression
Source: Int J Mol Sci. 2019 Aug 1;20(15):3713. doi: 10.3390/ijms20153713 (PMC6696180; doi:10.3390/ijms20153713)
Supplement: Supplementary file 1 [file ijms-20-03713-s001.zip › ijms-550517-supplementary/Supplementary Methods.docx]

**Supplementary Methods**

***Construction of LINC01537 stably overexpression cells***

Whole LINC01537 cDNA was synthesized with *BclI* and *XbaI* restriction sites adding at 5’ end and 3’ end, respectively. The cDNA was then cloned into the lentiviral expression vector pEZ-Lv201 (Genecopoeia Biotech Co.Ltd, Guangzhou, China). The vector carrying LINC01537 cDNA was further confirmed by direct sequencing. The vector together with the replication-defective VSV-G pseudotyped viral particles was co-transfected and packaged in 293T cells by a 3-plasmid transient cotransfection method with the Lenti-T HT packaging mix (Clonetech, US). The viruses were then harvested and concentrated. For transfection, A549 and PC-9 cells were infected with pEZ-Lv201- LINC01537 or empty pEZ-Lv201. After 48 h of transfection, the cells were stably selected with G418 at 100 μg/mL (Gibco, Lyon, France), and the drug-resistant cells were used for subsequent studies.

***Flow cytometry assay (FCM)***

Cells were collected with EDTA digestion and washed 2–3 times by pre-chilled phosphate buffer saline (PBS). Then the cell pellets were fixed by pre-chilled 70% ethanol overnight at 4 ℃. The fixed cells were subsequently washed 2 times by pre-chilled PBS, centrifuged to discard supernatants, resuspended in 500 μL propidium iodide staining solution, and incubated at room temperature for 30 min in the dark. 2 × 10^4^ to 3 × 10^4^ cells were finally submitted to FCM on FACScan (BD Biosciences, New York, USA) and cell cycle was analyzed using software CellQuest software (BD Biosciences). Furthermore, the fixed cells were resuspended in 1× Binding buffer with a concentration of 1.0 × 10^6^ cells/mL. 100 μL cell suspension was stained with 5 μL FITC-AnnexinV and 5 μL PI and then submitted to the FCM to determine cell apoptosis. Each experiment was performed in triplicate.

***Transwell assay***

For cell migration test, 2×10^4^ cells cultured in serum-free RPMI 1640 medium were seeded in the upper chamber of 8 μM Corning transwell insert chamber (New York, USA). Cell-free RPMI 1640 medium with 10% FBS was added to the lower compartment. After routine culture for 24 hours, the chamber was gently rinsed by PBS and cells in the upper membrane of chamber were scraped off using a cotton swab. Cells in lower surface of the membrane were fixed with 4% paraformaldehyde solution and stained with crystal violet (Invitrogen). Ten fields were randomly selected under a 100× microscope and number of cells that migrated to the lower layer was counted. Generally, the protocol for cell invasion test was similar as above except for the difference that the chamber was Matrigel-coated and the culture time was 48 hours.

***Plate clone formation assay***

Two hundred cells were inoculated into a dish containing 10 mL RPMI 1640 medium and gently rotated to evenly disperse the cells. The dishes were placed in the CO_2_ incubator for 2–3 weeks until visible colonies appeared. The colonies were fixed and stained with crystal violet, followed by air-drying, photographing, and counting.

***Cisplatin IC50 identification***

Cells were treated with cisplatin (20 μg/mL, 10 μg/mL, 5 μg/mL, 2.5 μg/mL, and 1 μg/mL). The cell viability was examined 24 hours later by routine Cell Counting Kit-8 (CCK8) assay. IC50 of cisplatin was calculated according to the dose-response curve.

***FISH***

Probe for LINC01537 was designed and synthesized by Ribo^TM^ Company (Guangzhou, China). FISH was performed with Ribo^TM^ FISH Kit according to the operating manual. In brief, cells were washed softly in PBS for 5 min and fixed in 4% formaldehyde for 10 min at room temperature. Then the cells were permeabilized in PBS containing 0.5% TritonX-100 for 5 min at 4 °C and washed with PBS 3 × 5 min. Hybridization was carried out with the probe in a moist chamber at 37 °C in the dark overnight. Then the cells were washed in PBS 3 × 5 min at 42 °C in the dark, stained with DAPI for 10 min and washed again in PBS. The cells were obtained and fixed on a load-bearing slide with a sealing tablet and photographed with a fluorescence confocal microscope.

***Cytosolic/nuclear fractions test***

Cytosolic and nuclear fractions were collected using nuclear/cytoplasmic isolation kit (Biovision, San Francisco, CA) according to the operating manual. LINC01537 expression was determined by qPCR.

***Ribonuclease Protection Assay (RPA)***

Total RNA was isolated from A549 and PC9 cells. After incubation at 37°C for 1 h, the RNA was digested with ribonuclease A+T at 37°C for 30 min. Then the reaction was terminated by incubation with proteinase K and 20% SDS at 37°C for 30 min. RNA was extracted by phenol-chloroform precipitation and analyzed by PCR using the primers: 5’-TTA CCC ATT CCT GCA GCC AG-3’ (Forward) and 5’- CCC ACA ACG CAC ACA GAA TG-3’ (Reverse). The PCR product was checked by electrophoresis on 1.5 % agarose gel.

***mRNA stability test***

Both A549 and PC9 cells were seeded at 24-well plate at 5×10^4^ per well. After 24 hours, the cells were treated with 2 mg/L actinomycin D. Then the cells were harvested in followed 30 minutes, 1 hour, 2 hours, 3 hours, and 4 hours, respectively. Total RNA was extracted and PDE2A expression was measured by qPCR.
