# Supplementary material for: Analysis of Survival-Related lncRNA Landscape Identifies A Role for LINC01537 in Energy Metabolism and Lung Cancer Progression
Source: Int J Mol Sci. 2019 Aug 1;20(15):3713. doi: 10.3390/ijms20153713 (PMC6696180; doi:10.3390/ijms20153713)
Supplement: Supplementary file 1 [file ijms-20-03713-s001.zip › ijms-550517-supplementary/Supplementary Table S1.docx]

**Supplementary Table S1.** Primers for SYBR-Green qPCR to determine expressions of candidate genes.

| Gene | Primers |
| --- | --- |
| *LINC01537* | 5’-TTGTGCAGAGGAAGCTCTCAG -3’(Forward) |
|  | 5’-TCCGCCTTTGTTTTCCTTCC -3’(Reverse) |
| *PDE2A* | 5’-CCTGCGCCTTCAACAAGCTA -3’(Forward) |
|  | 5’-GGCCTCCGTGATGATCTCCT -3’(Reverse) |
| *GAPDH* | 5’-ACAACTTTGGTATCGTGGAAGG -3’(Forward) |
|  | 5’-GCCATCACGCCACAGTTTC -3’(Reverse) |
| GLUT1 | 5’-CCGCAACGAGGAGAACCG -3’(Forward) |
|  | 5’-GTGACCTTCTTCTCCCGCATC -3’(Reverse) |
| HK2 | 5’-GAATGGGAAGTGGGGTGGAG-3’(Forward) |
|  | 5’-GAGGAGGATGCTCTCGTCCA-3’(Reverse) |
| PGK1 | 5’-CCACTGTGGCTTCTGGCATA-3’(Forward) |
|  | 5’-ATGAGAGCTTTGGTTCCCCG-3’(Reverse) |
| PKM2 | 5’-ACGAGAACATCCTGTGGCTG-3’(Forward) |
|  | 5’-AGGAAGTCGGCACCTTTCTG-3’(Reverse) |
| PDK1 | 5’-GGTGTTTACCCCCCTATTCAAG-3’(Forward) |
|  | 5’-CGGGAGGTCTCAACACGA-3’(Reverse) |
| LAHA | 5’-AGCCCGATTCCGTTACCT-3’(Forward) |
|  | 5’-CACCAGCAACATTCATTCCA-3’(Reverse) |
| G6PDH | 5’-TGGAGATCATCATGAAAGAGACC-3’(Forward) |
|  | 5’-GCGAATGACACCGTACTCCT-3’(Reverse) |
| DPYSL4 | 5’-AGAAAACCTCATCGTCCCTG-3’(Forward) |
|  | 5’-AGATCATGGTGGTTCCTCCT-3’(Reverse) |
| GLS2 | 5’-TGCCTATAGTGGCGATGTCTCA-3’(Forward) |
|  | 5’-GTTCCATATCCATGGCTGACAA-3’(Reverse) |
| L32 | 5’-TTCCTGGTCCACAACGTCAAG-3’(Forward) |
|  | 5’-TGTGAGCGATCTCGGCAC-3’(Reverse) |
| p21/CDKN1A | 5’-GTTCCTTGTGGAGCCGGAGC-3’(Forward) |
|  | 5’-GGTACAAGACAGTGACAGGTC-3’(Reverse) |
| MCP1 | 5’-ATCAATGCCCAGTCACCT-3’(Forward) |
|  | 5’-CTTTGGGACACTTGCTGCT-3’(Reverse) |
| IFNg | 5’-GGAGACCATCAAGGAAGACA-3’(Forward) |
|  | 5’-TGCTTTGCGTTGGACATTCA-3’(Reverse) |
| ALDOA | 5’-ATGCCCTACCAATATCCAGCA-3’(Forward) |
|  | 5’-GCTCCCAGTGGACTCATCTG-3’(Reverse) |
