# Supplementary material for: Analysis of Survival-Related lncRNA Landscape Identifies A Role for LINC01537 in Energy Metabolism and Lung Cancer Progression
Source: Int J Mol Sci. 2019 Aug 1;20(15):3713. doi: 10.3390/ijms20153713 (PMC6696180; doi:10.3390/ijms20153713)
Supplement: Supplementary file 1 [file ijms-20-03713-s001.zip › ijms-550517-supplementary/Supplementary Table S2.docx]

**Supplementary Table S2.** The base sequences for siRNAs.

| Gene | ID | Sense (5’-3’) |
| --- | --- | --- |
| *PDE2A* | siRNA-1 | GGAGGACAATCTCCAGCTT |
|  | siRNA-2 | GCAAGGTCATCGGAGACAA |
|  | siRNA-3 | GGTGGATGATGAGAGCTAT |
|  | siRNA-4 | GCTGGTGAACAAGATCAAT |
| *Negative control* | SiRNA-NC | UUCUCCGAACGUGUCACGUTT |
