# Supplementary figures and images for: Analysis of Survival-Related lncRNA Landscape Identifies A Role for LINC01537 in Energy Metabolism and Lung Cancer Progression
Source: Int J Mol Sci. 2019 Aug 1;20(15):3713. doi: 10.3390/ijms20153713 (PMC6696180; doi:10.3390/ijms20153713)

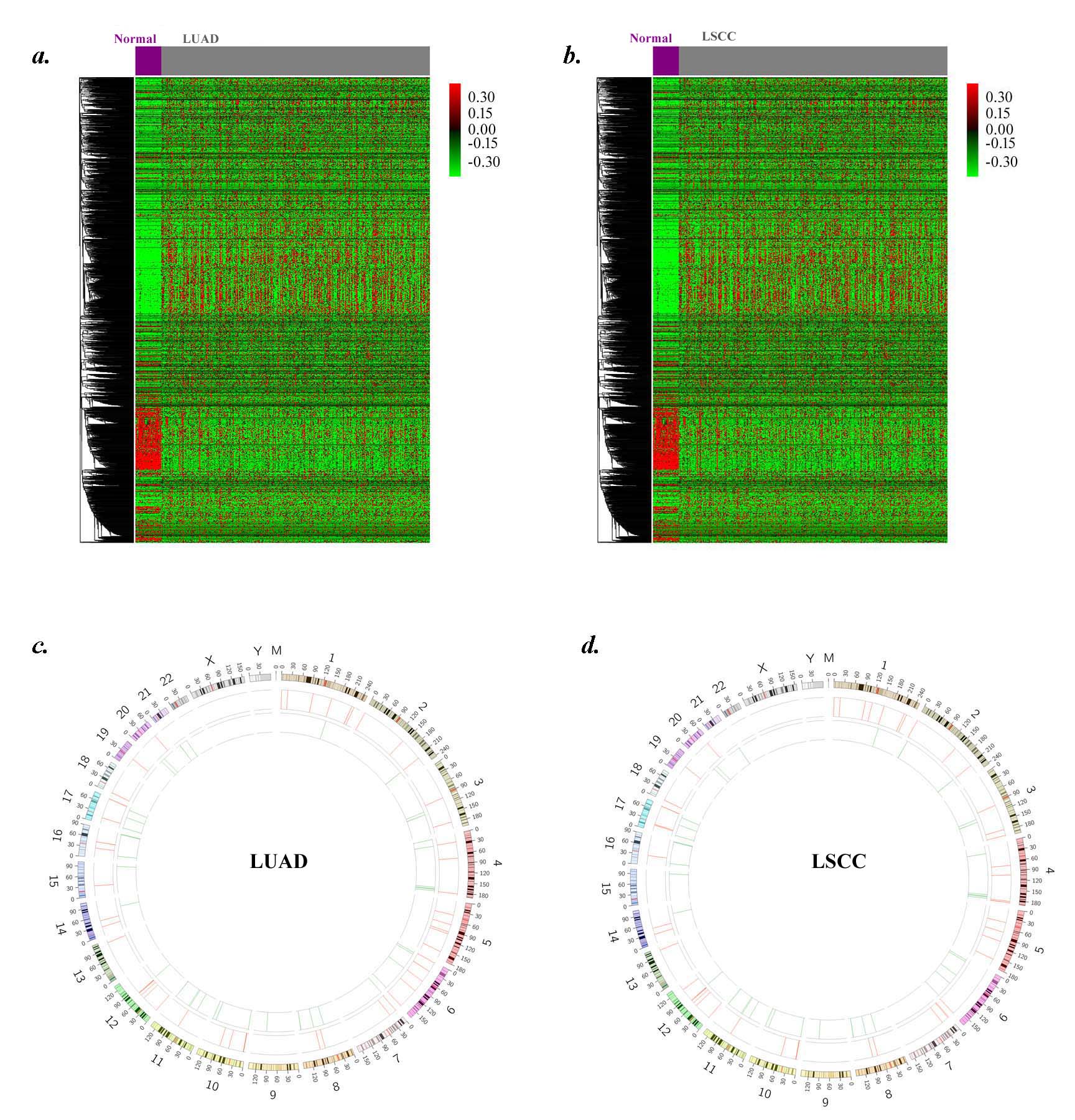

Supplement: Supplementary file 1 [file ijms-20-03713-s001.zip › ijms-550517-supplementary/Supplementary Figure S1.jpg]

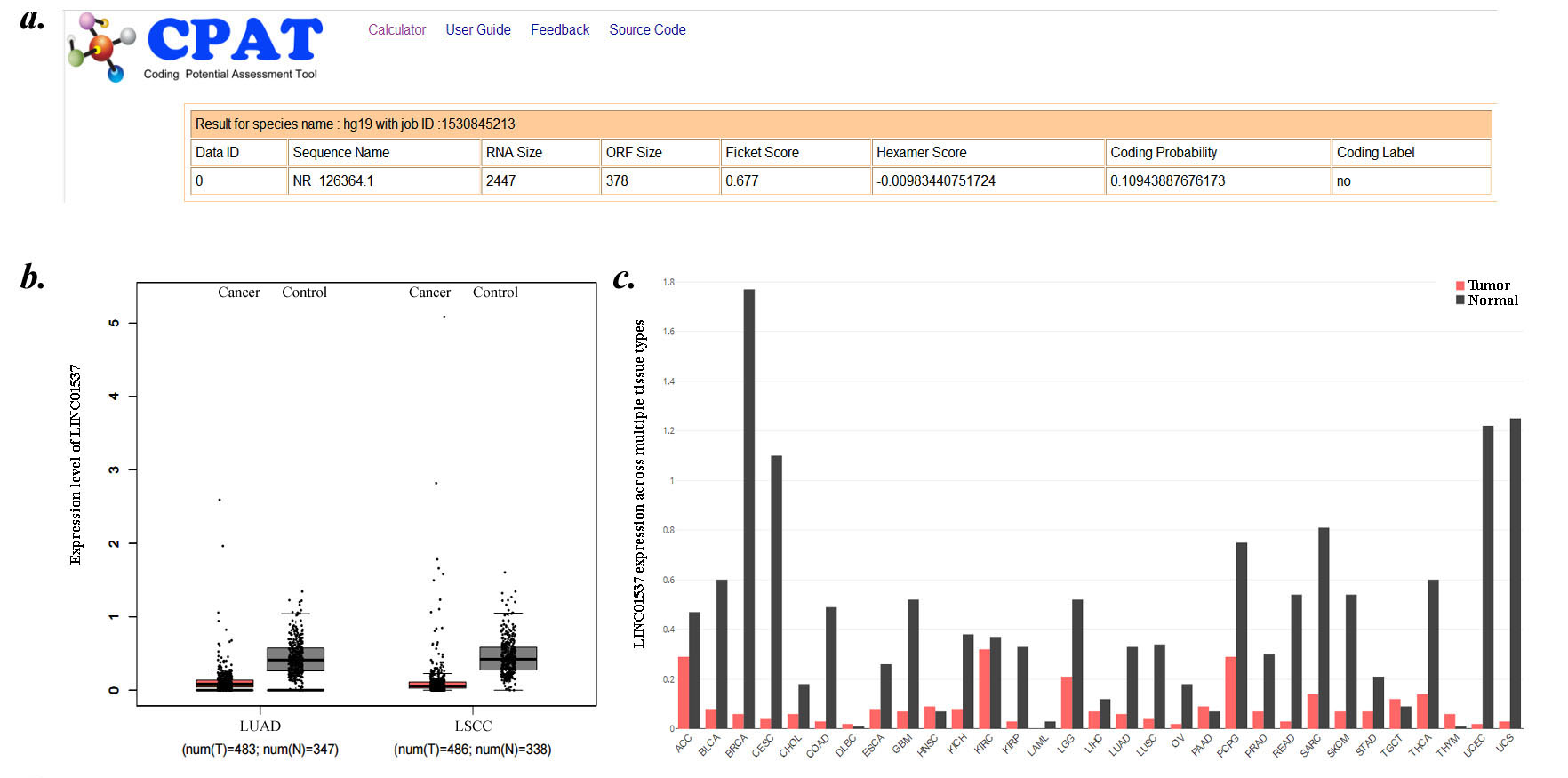

Supplement: Supplementary file 1 [file ijms-20-03713-s001.zip › ijms-550517-supplementary/Supplementary Figure S2.jpg]

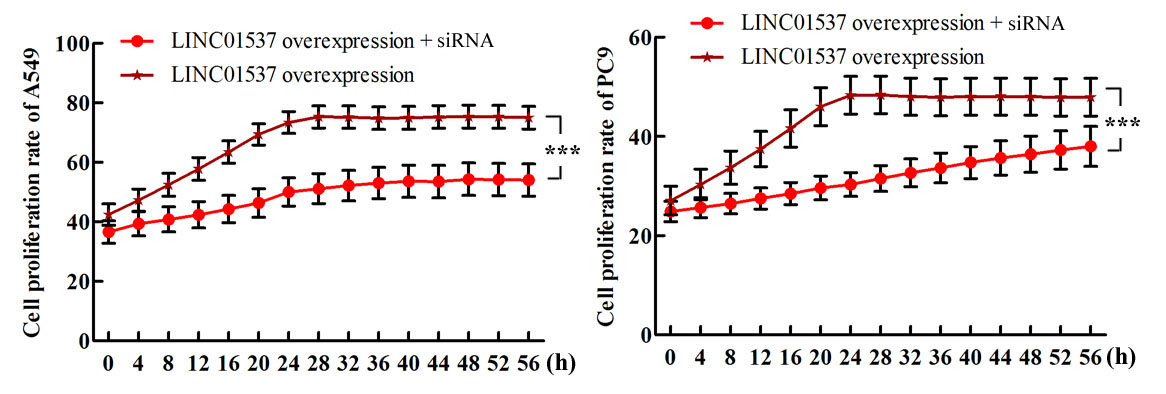

Supplement: Supplementary file 1 [file ijms-20-03713-s001.zip › ijms-550517-supplementary/Supplementary Figure S3.jpg]
